# The transcontinental variability of nonalcoholic fatty liver disease

**DOI:** 10.20517/2394-5079.2020.73

**Published:** 2020-10-12

**Authors:** Claudia P. Oliveira, Angelo Paredes, Mohammed Siddiqui, Lawrence Serfaty, Abhijit Chowdhury, Jose Tadeu Stefano, Denise Siqueira Vanni, Sherry Boyett, Arun J. Sanyal

**Affiliations:** 1 Department of Gastroenterology, Faculdade de Medicina da Universidade de São Paulo, São Paulo, SP 01000-000, Brazil; 2 Laboratório de Gastroenterologia Clínica e Experimental (LIM-07) do Departamento de Gastroenterologia e Hepatologia do Hospital das Clínicas HCFMUSP da Faculdade de Medicina da Universidade de São Paulo, São Paulo, SP 01000-000, Brazil; 3 Internal Medicine, Gastroenterology and Hepatology Services, Brooke Army Medical Center, San Antonio, TX 78234, USA; 4 Div.of Gastroenterology, Hepatology and Nutrition, Dept. of Internal Medicine, Virginia Commonwealth University School of Medicine, Richmond, VA 23298-0341, USA; 5 Viral and Metabolic Liver Unit, Department of Hepatology, Saint-Antoine Hospital, 75012 Paris, Paris; 6 Department of Hepatology, School of Digestive and Liver Disease, Institute of Post Graduate Medical Education & Research, Kolkata 700020, India; 7 Disease Biology, John C Martin Centre for Liver Research and Innovations Sonarpur, Kolkata 7000150, India

**Keywords:** Non-alcoholic fatty liver disease, phenotype, epidemiology, demographics

## Abstract

**Aim:**

To compare the phenotype of lean versus overweight (OW) and obese (OB) subjects with non-alcoholic fatty liver disease (NAFLD) across multiple continents.

**Methods:**

A retrospective study of histologically defined subjects from a single center each in France (Fr), Brazil (Br), India (In) and United States (US) was performed.

**Results:**

A total of 70 lean [body mass index (BMI) < 25 kg/m^2^] subjects (Fr:Br:In:US: 16:19:22:13) with NAFLD were compared to 136 OW (BMI > 25 kg/m^2^, BMI < 29 kg/m^2^) (*n* = 28:33:52:23) and 224 OB subjects (BMI > 29 kg/m^2^) (*n* = 81:11:22:103). Lean French subjects had the lowest incidence of type 2 diabetes while those from Brazil (*P* < 0.01) had the highest. Lean subjects had similar low-density lipoprotein-cholesterol, but higher high-density lipoprotein-cholesterol compared to obese subjects in all regions. In both lean and obese subjects, there were both insulin-sensitive and insulin-resistant subjects. Lean French subjects were most insulin-sensitive while those from Brazil were mostly insulin-resistant. For each weight category, subjects from India were more insulin-sensitive than those from other regions. Disease activity increased from lean to overweight to obese in France but was similar across weight categories in other regions.

**Conclusion:**

The phenotype of NAFLD in lean subjects varies by region. Some obese subjects with NAFLD are insulin-sensitive. We hypothesize that genetics and region-specific disease modifiers account for these differences.

## INTRODUCTION

Non-alcoholic fatty liver disease (NAFLD) is a major cause of liver-related morbidity and mortality^[1]^. Nonalcoholic steatohepatitis (NASH), the more aggressive form of NAFLD, can progress to cirrhosis and end-stage liver disease more frequently than non-alcoholic fatty liver (NAFL)^[2]^. The principal risk factors for NAFLD include obesity and insulin resistance (IR) and its associated conditions such as type 2 diabetes^[3–5]^.

Numerous reports from Asia and other parts of the developing world have identified NAFLD, including NASH, in lean subjects without biochemically obvious IR^[6,7]^. NAFLD has also been described in lean subjects in western countries^[8–10]^. There is however only limited information on the clinical, laboratory, and histological profile of lean versus obese subjects with NAFLD in the West. It is also not known if the histological spectrum of NAFLD and its relationship to IR is similar from one continent to another when subjects of identical body mass index (BMI) are considered. Similarly, when considering afflicted individuals with similar degree of IR, the distribution of histological findings from one continent to another is not known. These are likely to be relevant for either continuing a “one set of risk factors fits all” paradigm globally or informing development of “region-specific” risk stratification approaches.

In this study, we report data from four cohorts of subjects from the USA, Brazil, France and India to address the trans-continental drifts in disease phenotype across the spectrum of BMI and IR. The specific aims were to: (1) define the similarities and differences in disease expression between lean *vs*. obese subjects with NAFLD in the countries represented in this study; and (2) to compare the disease phenotype within specific weight and IR strata from one country to the next.

## METHODS

The current study is a retrospective cross-sectional analysis of a cohort of subjects with NAFLD at the participating centers. The participating centers were in Virginia, USA (PI: AJS), Brazil (PI: CPO), France (PI: LS) and India (PI: AC). The data sheets were developed at EUA: (2008-2013); France: (1999-2014); Brazil: (2009-2014); India (2008-2014); and analyzed by the investigators. The study involved an anonymized data set from an existing larger data set and was therefore considered exempt from IRB review.

### Patient population

Subjects with biopsy proven NAFLD with a full set of histological and laboratory data were included in this analysis from each site. Exclusion criteria included age < 18 years, absence of full data set including measures of IR, liver injury and function, anthropometric data, lipid profile and liver histology. Subjects with concomitant presence of alternate causes of liver disease, e.g., hepatitis C were also excluded to avoid their confounding effects. Finally, those with drug-induced NAFLD, TPN-associated NAFLD, and bariatric surgery within last 5 years or known infectious, e.g., HIV or known genetic disorders, e.g., abetalipoproteinemia associated with NAFLD, were excluded to keep the analysis focused on “garden-variety” NAFLD.

### Assessment of NAFLD

The liver histology was read by an experienced hepatopathologist at each site with multiple prior publications related to NAFLD. The presence of NAFLD was defined by the presence of hepatic steatosis (> 5%) confirmed by a liver biopsy in all instances; the nonalcoholic nature was established by clinical assessment of alcohol consumption to be less than 2 units daily for women and 3 units daily for men over the last 2 years^[11]^. Steatohepatitis was defined by the presence of steatosis (> 5%) along with lobular inflammation and cytological ballooning^[11]^. The severity of individual histological features of NAFLD was scored using the NIDDK NASH CRN criteria^[12]^.

### Assessment and stratification of body mass index

Lean, overweight and obese states were defined according to the World Health Organization and Modified National Cholesterol Education Program, Adult Treatment Panel III guidelines^[13]^. For subjects from India, two separate analyses were performed. In the first, the same body mass index cutoffs used in the West were used to define these states to directly relate the clinical-laboratory-histological findings across continents matched for BMI. A second analysis using the thresholds used for the Asian subcontinent was used in order to evaluate those who were lean versus obese using physiologically relevant parameters for the region^[14,15]^.

### Assessment and stratification of IR

IR was derived from fasting blood glucose and insulin. The Homeostatic Model of Assessment for IR (HOMA-IR) was calculated from the formula: [22.5 × fasting insulin (mU/mL) × glucose (mmol/L)]^[16]^.

To further visualize the relationship of glucose and insulin, the plasma insulin was plotted as a function of fasting plasma glucose. The resulting plot was divided in to four quadrants based on a fasting glucose threshold of 100 mg/dL and fasting insulin of 12 μU/mL. A fasting glucose below 100 mg/dL represents euglycemia and a fasting insulin level above 12 μU/mL has been associated with IR^[17,18]^.

Subjects with a glucose < 100 mg/dL and a fasting insulin < 12 μU/mL were considered insulin sensitive. Those with a glucose < 100 mg/dL but insulin levels > 12 μU/mL were considered to have IR with relatively intact beta cell function. Those with higher plasma glucose (> 100 mg/dL) and insulin > 12 μU/mL were considered to have severe IR. When the fasting plasma glucose levels were higher than 100 mg/dL and the corresponding insulin levels declined below 12 μU/mL subjects were considered to have advanced IR with beta cell failure. Type 2 diabetes was defined by a fasting blood glucose > 126 mg/dL in patients who had a hemoglobin A1c level of 6.5% or greater, an FPG level of 126 mg/dL or greater^[19]^.

### Plan of analysis

Data were analyzed using SPSS version 2. Descriptive statistics of subjects from each site was performed. Data for lean versus overweight versus obese subjects were compared for each country individually. Numerical data were compared using analysis of variance for normally distributed data. Quantitative variables with asymmetric distribution were described as median and interquartile range and compared between groups using the Kruskal-Wallis test. Next, subjects within each BMI strata from each country were compared to other countries correcting for multiple comparisons using Tukey’s test. A similar analysis of overweight or obese subjects was also performed. In another analysis, the cohort was stratified by the HOMA scores of < 2, 2-4 and > 4 and a similar analysis performed. Finally, a multivariable regression analysis was performed to evaluate the interactions between BMI, IR and other clinical parameters on liver histology in lean subjects with NAFLD in each region. Significance was set at a *P* value of 0.05.

## RESULTS

A total of 430 subjects with biopsy-proven NAFLD was enrolled (USA:Brasil:France:India 139:125:96:63) [Table 1]. They included 70 lean (BMI < 25 kg/m^2^) subjects (Fr:Br:In:US 22:16:19:13), 136 overweight (Is this BMI > 25 kg/m^2^, BMI < 29 kg/m^2^) (*n* = 52:28:33:23) and 224 obese subjects (BMI > 29 kg/m^2^) (*n* = 22:81:11:103). Subjects in the Indian and French cohorts were younger (mean 38.06 ± 1.6; 49.7 ± 1.2, respectively) compared to those from US and Brazil (mean 53.3 ± 0.92; 55.08 ± 0.2, respectively). In France about 70% of the subjects were male while in Brazil and USA approximately 70% were female. In the Brazilian cohort the prevalence of type 2 diabetes mellitus (T2DM) and hypertension were globally higher than other countries.

### Comparison of lean *vs*. overweight and obese subjects

#### Demographic and clinical profiles

**Figure 1.**
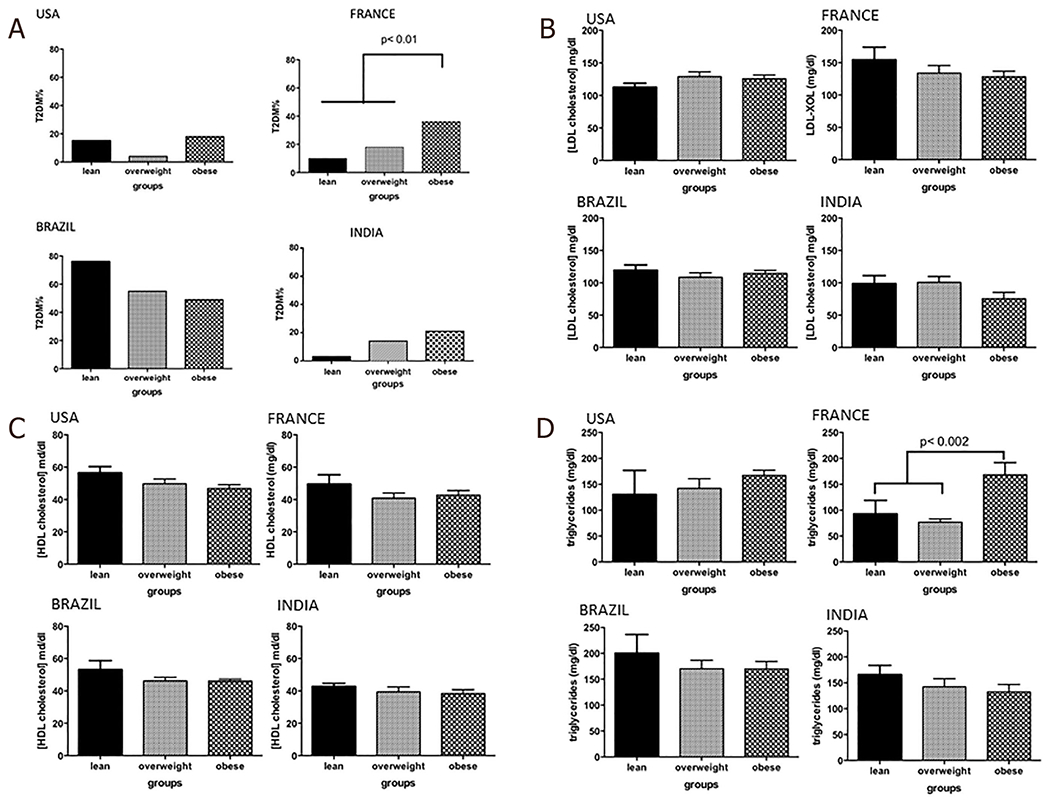
Comorbidities of lean *vs*. overweight *vs*. obese subjects with nonalcoholic fatty liver disease. A: type 2 diabetes mellitus (T2DM); B: LDL cholesterol; C: HDL cholesterol; D: triglycerides. LDL: low-density lipoprotein cholesterol; HDL: high-density lipoprotein cholesterol

In the US cohort, obese subjects were younger than overweight and lean subjects, respectively (51.9 ± 1.0; 53.3 ± 2.1; 61.8 ± 1.9; *P* = 0.02) [Table 1]. While the proportion of subjects with hypertension or requiring lipid-lowering therapy were similar across the different weight strata, overweight subjects had less type 2 diabetes compared to lean and obese subjects (3.7% *vs*. 22.2% *vs*. 28.4%, *P* = 0.01). In the Brazilian cohort the prevalence of T2DM was high in lean subjects, approaching 66% [Figure 1]. In the French cohort, the proportion of individuals with features of the metabolic syndrome increased progressively from lean to overweight to the obese groups. The Indian cohort had more males in the lean group (*P* < 0.001 *vs*. other groups) and had a progressively greater proportion of subjects with T2DM with progressively higher weight strata.

#### Insulin resistance

**Figure 2.**
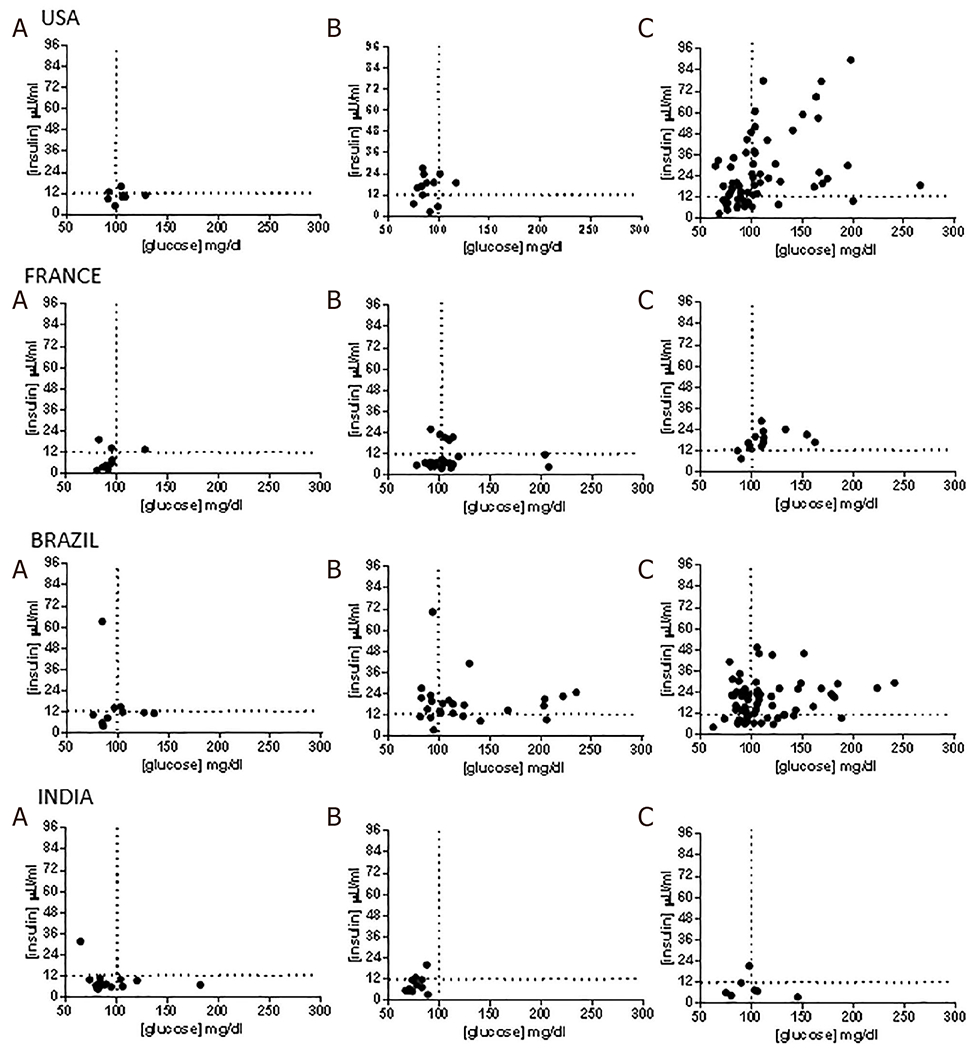
Insulin resistance and insulin sensitivity in nonalcoholic fatty liver disease lean subjects *vs*. overweight *vs*. obese across regions. A: lean subjects; B: overweight subjects; C: obese subjects

The distribution of insulin and fasting glucose values yielded interesting insights in all regions [Figure 2]. In the US, approximately 20% of lean subjects had a fasting blood glucose < 100 mg/dL and a fasting insulin less than 12 mIU/mL. The remaining subjects had evidence of increasing IR with 4 subjects demonstrating IR with beta cell failure, i.e., low fasting insulin (< 12 mIU/mL) despite a fasting glucose > 100 mg/dL. As expected, obese subjects had a substantially greater number of insulin resistant subjects with and without beta cell dysfunction. Interestingly, 11 (11.45%) obese subjects had both low fasting glucose and insulin levels suggesting that they were relatively insulin sensitive.

In the Brazilian cohort, 4 (25%) lean subjects had relatively low fasting insulin and glucose levels while the rest showed IR with or without beta cell failure [Figure 2]. As noted in the US cohort, a subset of subjects in the overweight and obese categories also were relatively insulin sensitive (fasting plasma glucose < 100 mg/dL, fasting plasma insulin < 12 μU/mL). In France, the majority of lean subjects were relatively insulin sensitive and IR increased progressively from lean-overweight-obese subjects and most overweight and obese subjects have more advanced IR. Subjects from India had lower fasting insulin levels compared to the other cohorts especially those from the US and Brazil even amongst obese subjects. Lean and obese subjects in the Indian cohort had similar insulin sensitivity.

#### Histology in lean versus obese subjects

**Figure 3.**
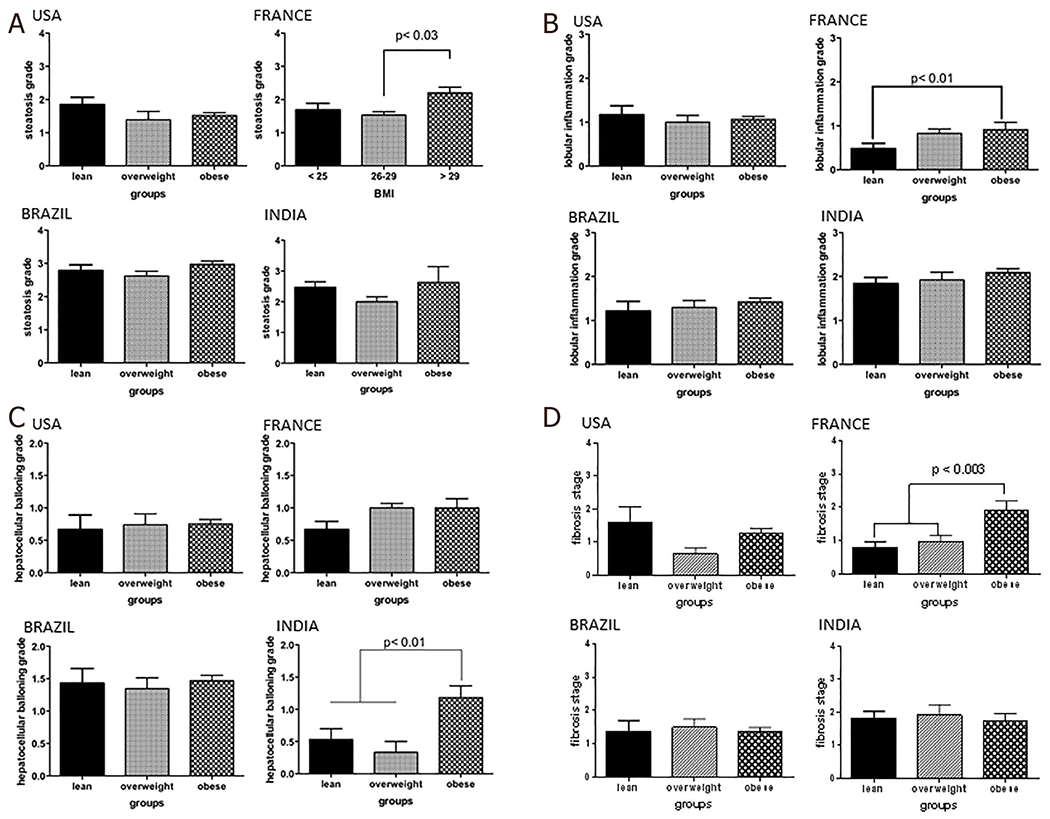
The histological spectrum of nonalcoholic fatty liver disease in lean *vs*. overweight *vs*. obese across regions. A: steatosis grade; B: lobular inflammation grade; C: hepatocellular ballooning; D: fibrosis stage

The steatosis grade was similar across lean, overweight, and obese subjects in the US cohort [Figure 3]. Similarly, the inflammation, ballooning grades and fibrosis stage were similar across the weight strata. The spectrum of liver histology in subjects from Brazil was similar to that seen in the US. Importantly, the severity of steatosis, inflammation and ballooning were similar across the three weight strata in this country.

On the other hand, obese subjects in the French cohort had significantly greater steatosis grade compared to both lean and overweight subjects (*P* < 0.002). There was also a stepwise increase in inflammation grade from lean to overweight to obese subjects which reached significance (lean *vs*. obese *P* < 0.01). There was a trend for lower ballooning scores in the lean subjects (*P* = 0.06). Lean and overweight subjects also had a significantly lower fibrosis stage compared to the obese subjects (*P* < 0.01 for both).

Lean Indian subjects had similar degrees of steatosis compared to overweight and obese subjects. The lobular inflammation grades were also similar across the three weight groups. However, in contrast to other regions, the ballooning grade was significantly lower in lean and overweight subjects compared to obese subjects (*P* < 0.01 for both). The fibrosis stage was similar across the three weight categories. Recalibrating BMI threshold for Indians to 22 kg/m^2^ for the diagnosis of obesity did not alter the results qualitatively.

### Clinical-laboratory-histological variability within similar weight strata

#### Lean subjects

Lean subjects in the US were also more likely to be hypertensive (55.6%) compared to those from Brazil (33.3%), France (14.3%), and India (15.8%). Only 9.5% of lean US subjects had T2DM compared to 26% of subjects in India and 66% of subjects in Brazil [Table 2]. There were no differences in the lipid profile of lean subjects across the various cohorts. However, subjects in Brazil were more insulin resistant than those in the other groups.

In line with greater IR, lean subjects from Brazil (mean grade 2.8) had a higher steatosis grade (*P* < 0.0005) compared to subjects from the US (mean grade 1.9), France (mean grade 1.8), and India (mean grade 2.2). Lean subjects from Brazil also had greater lobular inflammation (mean grade 1.5) compared to subjects from the other countries (mean grade range 0.5-0.7) (*P* < 0.0001). The ballooning grade was similar across the US, Brazilian, and Indian cohorts but was lower in lean subjects from the French cohort (*P* < 0.0001). Similarly, the mean fibrosis stage was similar across the US, Brazil, and India but higher than that seen in the French group (*P* < 0.0013).

#### Overweight subjects

The prevalence of hypertension and T2DM was higher in the subjects from Brazil. The mean HOMA scores were similar across the four groups. As noted in the lean subjects, a proportion of subjects were still relatively insulin sensitive whereas a proportion was insulin resistant with beta cell failure. Subjects from Brazil had more advanced IR with a majority of subjects with a fasting plasma glucose > 100 mg/dL. Interestingly, the ballooning grade was more severe in overweight subjects from India compared to those from the USA and France (1.7 *vs*. 1 *vs*. 0.8, *P* < 0.0001). Subjects from Brazil had intermediate degrees of ballooning injury (mean grade 1.2). The fibrosis stage in overweight subjects in the Indian cohort was modestly higher than the other cohorts (mean: 1.8 *vs*. 0.6 *vs*. 1 *vs*. 1.2 India *vs*. US *vs*. France *vs*. Brazil) [Table 3].

#### Obese subjects

In Brazil almost 80% of the obese subjects were hypertensive while 49.5%, 44.4%, and 18.2% of obese subjects in US, France, and India respectively were hypertensive. 100% of the obese subjects in the Indian cohort had T2DM [Table 4]. The lipid profiles were comparable amongst obese subjects from different countries.

As expected, most subjects had IR. Interestingly, a subset of obese subjects from each country had a fasting plasma glucose < 100 mg/dL as well as a fasting plasma insulin < 12 μU/mL indicating that they were relatively insulin sensitive. The proportion of such individuals was greatest in the cohort from India. The US cohort had somewhat lower steatosis grade whereas subjects from India had more inflammation and ballooning. The fibrosis stages were similar across the groups.

### Transcontinental variability in histological severity in those with similar degrees of IR

A potential reason for the variability in histology from one country to another could be the variable degree of IR in each weight strata. In order to evaluate this, subjects were stratified into those with mild, moderate and severe IR (HOMA: 0-2, 2.1-4 and > 4 respectively). The severity of individual histological features was then assessed across the four study cohorts.

As expected, the severity of steatosis was largely similar across the four groups with the exception of somewhat lower steatosis in the Indian group in those with moderate IR (*P* < 0.01 *vs*. France). On the other hand, the group in Brazil had lower scores for lobular inflammation compared to other groups across multiple IR strata. They also had lower ballooning grades especially in those with moderate or severe IR. This was accompanied by less fibrosis across all strata of IR.

## DISCUSSION

Body weight and IR are two of the best-known risk factors for NAFLD. While it is known that many subjects with NAFLD in Asia are lean and do not have the usual biochemical features of IR, it was not known if a similar profile was also seen in lean subjects in other regions of the world. It is however generally believed that the relationship between body weight and severity of IR on one hand, versus liver histology is generally similar in all parts of the world. The current study challenges this “one size fits all” approach and demonstrates substantial differences and heterogeneity from countries in one continent versus even within similar weight strata.

The prevalence of obesity, T2DM and hypertension are higher in the USA and Brazil than in some European countries like France^[20–23]^. In Brazil, the prevalence of arterial hypertension found was 21.4% (95%CI: 20.8-22.0) using the self-reported criterion, 22.8% (95%CI: 22.1-23.4) for measured arterial hypertension, and 32.3% (95%CI: 31.7-33.0) for hypertension arterial pressure and / or report of medication use^[24]^. Furthermore, according IDF Atlas, in Brazil and USA, the prevalence of T2DM in 2019 was 8.5% and 11.1% respectively, while in Europe was 6.3%. USA is the third-highest country with patients with T2DM in the world, and Brazil the fifth^[20]^. Also, caloric consumption in American countries is higher than in some countries and in Europe^[25]^. In addition, patients in Brazil and the USA come from large tertiary-level university hospitals, where patients of greater severity are referred to them.

An important observation in this study is the remarkable range of laboratory-histological findings when comparing lean subjects with overweight and obese subjects in the different countries where this study was conducted. In France, a clear stepwise worsening of IR and severity of liver histology was noted with increasing BMI. In contrast, lean subjects in Brazil were more insulin-resistant and like overweight and obese subjects with respect to both the severity of their IR and liver histology. Subjects from the United States and India had an intermediate relationship with Indian subjects tending to demonstrate generally lower insulin levels than those from the other cohorts at all weight strata. These corroborate the concept that the severity of NAFLD is not a simple function of increasing body mass and that these relationships can be variable from one region to another.

The current study further demonstrates that the spectrum of IR and liver histology is variable from one country to another even when the body mass is accounted for. Lean subjects in the Brazilian cohort were more insulin-resistant and had greater steatosis than subjects from other regions. However, the severity of cytological ballooning was similar in the US, Brazil, and India and higher than that seen in the French subjects. These data are in line with the concept that development of liver injury is more than a simple function of IR and that additional factors are likely to play a role. While theoretically possible, we believe it is unlikely that these data purely represent differences in how the histology was read by the local pathologists since all of the pathologists involved are senior and experienced pathologists who have previously published in the field.

Another noteworthy finding in this study is that even in Western countries, a subset of overweight and even obese subjects with NAFLD were relatively insulin sensitive, i.e., with a fasting insulin < 12 μU/mL and [glucose] < 100 mg/dL. The lower blood glucose could not be attributed to the use of anti-diabetic therapy alone although it may have played a role in a few subjects. The only potential exception was the obese Indian cohort where all subjects were known to have diabetes and many also had a fasting plasma glucose < 100 mg/dL. While the presence of insulin sensitive obese individuals is well established^[26]^, there is a general perception that these so called “fit-fat” subjects do not develop end organ diseases typically associated with IR. The current study further demonstrates that the distribution of liver histology in these subjects includes the full spectrum of NAFLD. The mechanisms underlying the liver disease in these subjects are not well understood. Unfortunately, due to the retrospective nature of this current analysis, the subjects were not genotyped and their PNPLA3 and TM6SF2 and other SNPs associated with the metabolic syndrome were not available. This is now a logical future direction of research in the field.

It is also not known if obese insulin-sensitive subjects respond to insulin sensitizers and improve their liver histology in the same manner as obese insulin resistant subjects. In the PIVENS trial, baseline HOMA scores did not predict histological response to pioglitazone^[27,28]^. This study however did not evaluate assess the insulin sensitivity status by HOMA alone but by graphical analysis of the relationship of glucose and insulin. It is also recognized that an euglycemic hyperinsulinemic clamp is the gold standard for measurement of insulin sensitivity^[29]^. In this retrospective analysis, this was not possible. However, it will be valuable to perform these in obese subjects with NAFLD who have a low fasting glucose and insulin level in the future to be certain that they are indeed insulin sensitive. Regardless, more information about the pathophysiological mechanisms underlying disease development and progression in obese or overweight insulin sensitive subjects will help future efforts to tailor specific therapies that are most likely to engage the relevant therapeutic targets in these individuals.

There is a difference in the aggressiveness of NAFLD between American and European countries, mainly Mediterranean countries. One interesting example is the number of liver transplantations in USA secondary NASH compared with Europe countries^[30]^. In Brazil there has also been an increase in the numbers of liver transplantations secondary to NASH. Several hypotheses can be considered for the greater severity of fibrosis in American countries, including lean NASH: (1) Higher caloric intake in the Americas, diet rich in fructose and trans lipids^[31]^ low consumption of Mediterranean diet, including fiber and omega 3; (2) greater sedentary lifestyle in American countries; (3) presence of genetic factors such as PNPLA3^[32]^; and (4) dysbiosis, even in Lean NASH patients^[33]^. Furthermore, in the USA and Brazil, there is also admixture with genes from non-caucasian Hispanic and native American genes. American Hispanics have more severe NASH. The complex genetic background may be another potential explanation. Unfortunately given the sample size of this initial assessment, it is not feasible to address the role of genetics. Similarly, there may be differences in diet and also microbiome functionality that are not captured by current methods that could play a role. NASH is a complex disorder driven by gene environment interactions.

There were also differences in the spectrum of other end organ diseases within lean subjects with NAFLD. These corroborate the concept that the pathogenesis of each of these conditions is complex and there are regional variations in the prevalence rates and severity. All of these data further attest to the transcontinental drifts in phenotype development of this cluster of diseases.

There are several potential explanations for the observed variations in NAFLD phenotype and associated conditions across the four cohorts studied. These include genetic differences in susceptibility, regional variation in diet patterns, differences in the intestinal microbiome, exercise, environmental exposures etc. While a detailed analysis of these is beyond the scope of this manuscript, such studies are now indicated to better understand the variations in phenotype development in different regions.

Perhaps the most relevant implication of the current study is that it provides proof of concept that there are differences in both the histological phenotype and associated clinical features in subjects with NAFLD in different regions of the world and that region-specific data are now needed to provide optimal guidance for clinical care on various regions. Also, it raises the possibility that therapeutic responses in one region may or may not be similar from one region to another. A clearer understanding of variable disease mechanisms underlying the differences in phenotype development should help targeting therapeutics towards the most relevant targets in a given region and phenotype in the future.

The principal limitations of the current study are the relatively modest size of lean subjects with NAFLD in various regions and the potential for ascertainment bias. The small numbers of lean subjects however reflect the fact that they represent a small fraction of all subjects with NAFLD. Also, another limitation is that we report the prevalence of fibrosis stage in patients with NAFLD who had a biopsy at the centers in this study. Since this was done according to standard of care, the selection of patients for a biopsy was variable across centers. Further, the distribution of fibrosis could also be impacted by ascertainment bias due to the nature of the center. It was seen in the Indian cohort probably because increasing age is linked to higher stages if fibrosis and is generally considered to be due to longer exposure to disease state. It is potentially possible that the low fibrosis stage in India reflected the lower age of the population studied. It is noted in the section about variations across centers and as a potential explanation for the differences in fibrosis stage in India *vs*. other sites. However, most published studies of NAFLD also have the same ascertainment bias associated with tertiary care academic medical centers. These limitations notwithstanding, the current study demonstrates substantial variability in disease phenotype from one region to another.

In summary, the current study provides novel information on the variability in disease phenotype in lean as well as overweight and obese subjects in different parts of the world. It challenges the paradigm that all lean subjects with NAFLD have mild IR and have mild forms of liver disease. Conversely, it also demonstrates that a fraction of obese subjects with the full spectrum of NAFLD may be relatively insulin sensitive. Future studies to define the mechanistic basis for these differences may inform therapeutic choices in subjects in different regions.

## Figures and Tables

**Table 1.** Demographics and clinical profiles across-country in subjects with NAFLD

Parameter	USA	France	Brazil	India
Age (years)	53.3 ± 0.92	49.7 ± 1.2*	55.08 ± 0.2	38.06 ± 1.6*
Females (%)	71.2	30.9	46.5	66.2
T2DM (%)	21.1	21.6	53.3*	31.0
Hypertension (%)	43.7	25.3	67.4*	11.6
Triglycerides (mg/dL)	204.9 ± 10.3	112.5 ± 11.4*	218.0 ± 7.9	198.3 ± 12.4
Total Cholesterol (mg/dL)	197.8 ± 4.2	206.5 ± 5.5	193.5 ± 4.2	165.8 ± 7.7
LDL-XOL (mg/dL)	125.8 ± 4.5	135.6 ± 7.1	115.5 ± 3.2	93.3 ± 6.6
HDL-XOL (mg/dL)	44.0 ± 5.4	43.0 ± 5.2	46.0 ± 5.5	40.0 ± 4.7

* statistical significance. T2DM: type 2 diabetes mellitus; LDL: low-density lipoprotein cholesterol; HDL: high-density lipoprotein cholesterol; NAFLD: non-alcoholic fatty liver disease

**Table 2.** Comparison of lean subjects across study cohorts

Parameter	USA	Brazil	France	India
Age (years)	58.3 ± 0.7	57.7 ± 4	46 ± 6	40.5 ± 3
Males (%)	0	33.3	71.4	84.2
Type 2 diabetes (%)	22.2	55.6	9.5	26.3
Hypertension (%)	55.6	33.3	14.3	15.8
Dyslipidemia (%)	44.4	55.6	33.3	31.6
AST (IU/L)	50 (40-78)	35 (20-93)	29 (19-47)	45 (24-60)
ALT (IU/L)	49 (30-114)	52 (22-101)	60 (45-79)	56 (31-84)
GGT (IU/L)	170 (134-276)	50 (25-286)	75 (50-134)	45 (36-72)
Triglycerides (mg/dL)	83 (83)	129 (99-221)	52 (22-100)	132 (111-187)
HDL cholesterol (mg/dL)	47 (47)	51 (38-66)	44 (37-63)	43 (35-49)
INR	1 (0.9-1)	1 (1-1)	1.03 (1-1.10)	1.2 (1.17-1.22)
Steatohepatitis (%)	55.6	77.8	60	84
Steatosis grade - 0/1/2/3 (%)	0/33/44/23	0/22/55/22	0/55/20/25	10.5/36.8/47.4/5.3
Lobular inflammation - 0/1/2/3 (%)	12.5/62.5/12.5/12.5	33/22/33/12	50/40/10/0	26.3/63.2/10.5/0
Ballooning - 0/1/2 (%)	44.4/22.2/33.4	11/77/12	35/60/5	53/40/7
Fibrosis stage - 0-none/1-perisinusoidal/2-portal-periportal/3-bridging/4-cirrhosis (%)	11.1/33.3/0/33.3/22.2	0/44/44/12/0	21.4/50/14.3/14.3/0	57.9/15.8/15.8/10.5/0
Glucose (mg/dL)	95 (87-107)	88 (85-126)	90 (83-95)	84 (80-95)
Insulin (mU/mL)	10 (7-12)	11 (6-15)	5 (3-14)	7 (6-10)
HOMA	2.35	2.39	1.11	1.45

ALT: alanine aminotransferase; AST: aspartate aminotransferase; HOMA: homeostatic model assessment; INR: international normalized ratio

**Table 3.** Comparison of overweight subjects across study cohorts

Parameter	USA	Brazil	France	India
Age (years)	54.1 ± 6	55.1 ± 2	51 ± 7	34.1 ± 5
Males (%)	30	48.6	70.2	46.2
Type 2 diabetes (%)	3.7	56.8	19.3	14.3
Hypertension (%)	30	45.9	23.6	0
Dyslipidemia (%)	44.4	73.0	21.4	30.8
AST (IU/L)	50 (39-84)	30 (22-41)	31 (23-48)	40 (29-56)
ALT (IU/L)	60 (46-95)	36 (27-57)	63 (48-80)	45 (38-79)
GGT (IU/L)	83 (38-156)	66 (30-160)	50 (27-103)	46 (33-55)
Triglycerides (mg/dL)	141 (76-195)	150 (107-241)	66 (40-108)	120 (112-155)
HDL cholesterol (mg/dL)	50 (40-61)	48 (38-52)	40 (30-52)	37 (32-45)
INR	1 (1-1)	0.98 (0.95-1)	1.08 (1.02-1.13)	1.2 (1.14-1.27)
Steatohepatitis (%)	60	73	80	70
Steatosis grade - 0/1/2/3 (%)	25/30/15/30	5.4/40.5/43.2/10.8	0/50/40/10	15.4/69.2/15.4/0
Lobular inflammation - 0/1/2/3 (%)	20.8/58.3/20.8/0	14.3/54.3/25.7/5.7	19.6/64.3/14.3/1.8	23.1/61.5/15.4/0
Ballooning - 0/1/2 (%)	45.8/37.5/16.7	60/40/10	12.5/71.5/16	67/33/0
Fibrosis stage - 0-none/1-perisinusoidal/2-portal-periportal/3-bridging/4-cirrhosis (%)	0/57.7/19.4/15.4/7.7	36.1/13.9/27.8/17.7/5.6	26.2/35.7/19.0/7.1/11.9	53.8/7.7/30.8/7.7/0
Glucose (mg/dL)	91 (83-102)	110 (94-141)	103 (94-113)	78 (72-84)
Insulin (mU/mL)	16 (10-19)	16 (12-21.5)	7 (5-16)	7 (6-12)
HOMA	3.6	4.35	1.78	1.35

ALT: alanine aminotransferase; AST: aspartate aminotransferase; GGT: gamma glutamyl transferase; HOMA: homeostatic model assessment; HDL: high-density lipoprotein cholesterol; INR: international normalized ratio

**Table 4.** Comparison of obese subjects across study cohorts

Parameter	USA	Brazil	France	India
Age (years)	52.4 ± 2	55.1 ± 5	50.7	38.4 ±5
Males (%)	38	24.7	66.7	9.1
Type 2 diabetes (%)	28.4	51.9	44.4	100
Hypertension (%)	50	79.2	44.4	18.2
Dyslipidemia (%)	38	70.1	61.1	9.1
AST (IU/L)	40 (27-63)	35 (20-93)	48 (29-58)	36 (23-62)
ALT (IU/L)	53 (34-83)	52 (22-101)	76 (57-99)	33 (29-78)
GGT (IU/L)	38 (27-66)	53 (27-87)	55 (36-128)	33 (28-46)
Triglycerides (mg/dL)	149 (107-211)	129 (99-221)	141 (107-256)	126 (99-155)
HDL cholesterol (mg/dL)	43 (36-50)	51 (38-66)	43 (36-48)	38 (32-42)
INR	1 (0.97-1.0)	1 (1.0-1.0)	1.04(0.96-1.06)	1.3 (1.2-1.3)
Steatohepatitis (%)	56.8	89.6	81.3	90
Steatosis grade - 0/1/2/3 (%)	6.5/44.6/33.7/15.2	5.3/20.0/45.3/10.8	0/13/57/30	0/36.4/63.6/0
Lobular inflammation - 0/1/2/3 (%)	8.8/76.9/14.3/0	8/46.7/37.3/8	12.5/68.8/18.8/0	0/90/10/0
Ballooning - 0/1/2 (%)	38.6/45.5/15.9	46.6/52/1.3	12.5/68.8/18.8	9/63.6/27.4
Fibrosis stage - 0-none/1-perisinusoidal/2-portal-periportal/3-bridging/4-cirrhosis (%)	36.6/28.9/8.9/21.1/4.4	22.7/44/10.7/16/6.7	12.5/18.8/31.3/12.5/25.0	45.5/36.4/18.2/0/0
Glucose (mg/dL)	101 (86-124)	103 (90-127)	104 (96-143)	93 (81-106)
Insulin (mU/mL)	20 (14-37)	17 (11-26)	17 (14-20)	7 (5-20)
HOMA	4.99	4.32	4.37	1.65

AST: aspartate aminotransferase; ALT: alanine aminotransferase; GGT: gamma glutamyl transferase; HDL: high-density lipoprotein cholesterol; INR: international normalized ratio; HOMA: homeostatic model assessment

## Data Availability

Not applicable.
